# In-Process Atomic-Force Microscopy (AFM) Based Inspection

**DOI:** 10.3390/s17061194

**Published:** 2017-05-31

**Authors:** Samir Mekid

**Affiliations:** Mechanical Engineering Department, King Fahd University of Petroleum and Minerals, Dhahran 31261, Saudi Arabia; smekid@kfupm.edu.sa; Tel.: +966-13-860-7746

**Keywords:** in-process inspection, AFM, nanomachining, probes, nanoscale inspection

## Abstract

A new in-process atomic-force microscopy (AFM) based inspection is presented for nanolithography to compensate for any deviation such as instantaneous degradation of the lithography probe tip. Traditional method used the AFM probes for lithography work and retract to inspect the obtained feature but this practice degrades the probe tip shape and hence, affects the measurement quality. This paper suggests a second dedicated lithography probe that is positioned back-to-back to the AFM probe under two synchronized controllers to correct any deviation in the process compared to specifications. This method shows that the quality improvement of the nanomachining, in progress probe tip wear, and better understanding of nanomachining. The system is hosted in a recently developed nanomanipulator for educational and research purposes.

## 1. Introduction

Manufacture of small components with ever increasing accuracy is highly requested in the market of precision systems [1]. Some nanoscale features are machined with highly requested accuracy and are predicted to reach the subnanometer level in 2020 by Tanigushi [2]. The nanomachining and micromachining are however, associated with their own related phenomena. Effects of van der Waals force are more apparent at nanoscale, while adhesion between asperities, fracture, surface layer formation, and debris formation are important at microscale. It is known that phenomena of materials fracture are manifold since they are governed by the microscopic properties of a material that are varying from one material to another.

Although ultra-precision machines are designed to achieve this ultra-precision [3], machining conserves its share of errors and needs compensation in in-process. Hence, fabricating at nanoscale and microscale may not be very deterministic if the functions of manufacturing e.g., material removing is carried out, the part is removed, and the obtained features are inspected and expected to be very close to specifications. At this scale, it becomes important to inspect while in-process and correct if there is a need to stay within the specifications for the reasons discussed previously.

This approach of in-process inspection has been used in manufacturing for decades using different measurements techniques on machine tools for standard parts. A review has been carried out [4] for standard scale machines. Recent sensors have been proposed to measure specific features such as roundness [5,6], surface finish [7], and dimensional measurement [8]. The search of the public literature shows little contribution in the area of in-process inspection at nanoscale. The characterization of the nano-lithography is usually carried out by the same atomic-force microscope (AFM) probe used to remove material, showing quality measurement degradation [9] and using AFM for nano-patterning and imaging for mask repair based on the same principle [10]. Other work included multi pass scratching method with depth prediction using an AFM tip [11].

The AFM probe can be used for specific lithography work and retract back to inspect the work done, but this practice will degrade the probe tip and hence, affects the measurement quality. To avoid this, we propose in this paper to add a second probe for lithography work that is designed and assembled back-to-back to an AFM probe within one system. The AFM probe will keep its measurement quality without being disturbed by the other degrading tasks.

A new era has been triggered in engineering and science where material properties can be designed and controlled at the level of individual atoms and molecules. The proposed in-process inspection serves the following specific objectives:

(a) To provide nano-machining processes with in-process inspection using back-to-back probes one of which is AFMs.
(b) To build nano-devices with specific geometries and particular characteristics.
(c) To inspect and characterize complex shapes at corresponding accuracies.

## 2. Proposed Design System

### 2.1. Host System Description

**Figure 1:**
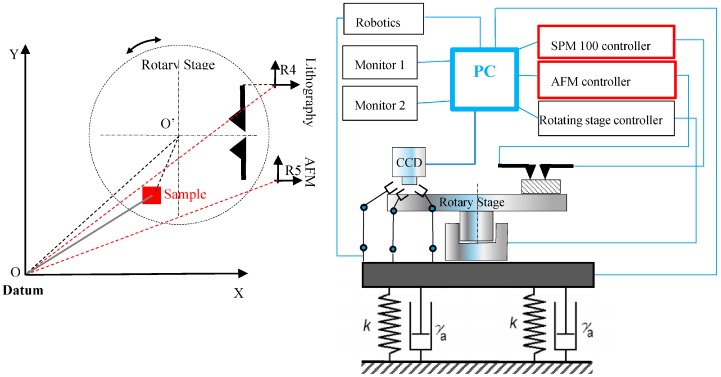
Schematic of the integrated manipulator and overall coordinate system.

**Figure 2:**
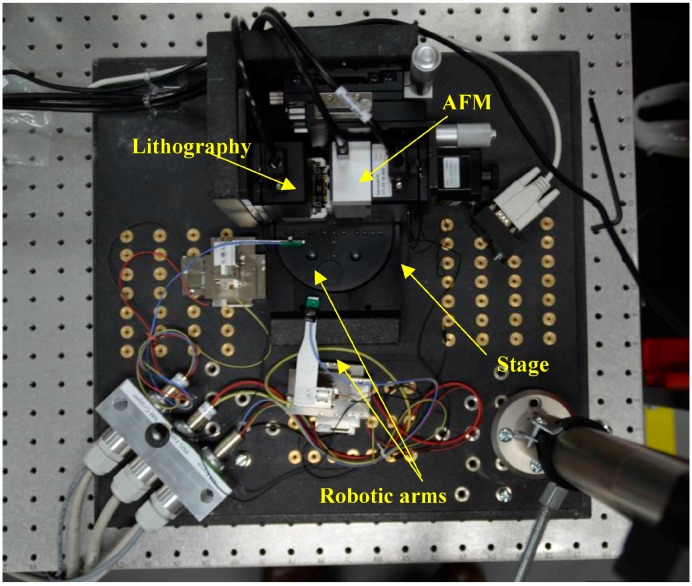
Nanomanipulator platform with nanomachining.

Based on the previous reviews and objectives, we propose a recently developed in-process inspection for nanomachining based on AFM probing system. The host system is a nanomanipulator having one side for sample preparation and robotics manipulation, and, the other side for nanomachining such as nanolithography with in-process inspection (Figure 1). This instrument encompasses nano-manufacturing with in-process inspection, assembly, and inspection/characterization of material particles or parts at the nanoscale. The instrument comprises all operations in one single rotating stage keeping the same datum for the samples that are under investigation. This rotating stage has three zones. The first one is the sample preparation zone. The second one is for samples manipulation and equipped with three robotic arms, each having three degrees of freedom (DOF) with a nanometer resolution over a 10 mm range of motion. This manipulation area is monitored via a CCD camera having a resolution of 1 µm. This can inspect samples and probes. The third zone has the AFM inspection with in-process (i.e., nanomachining) inspection. The nanomachining encompasses numerically controlled nanomachining e.g., lithography with its probe mounted back-to-back to an AFM probe for in-process inspection as a new feature. A suction device is added to clean the on-going machining debris. A top view of the whole instrument is shown in Figure 2. Most parts are homemade except technology hardware e.g., AFM, robotic arms, rotating stage.

**Figure 3:**
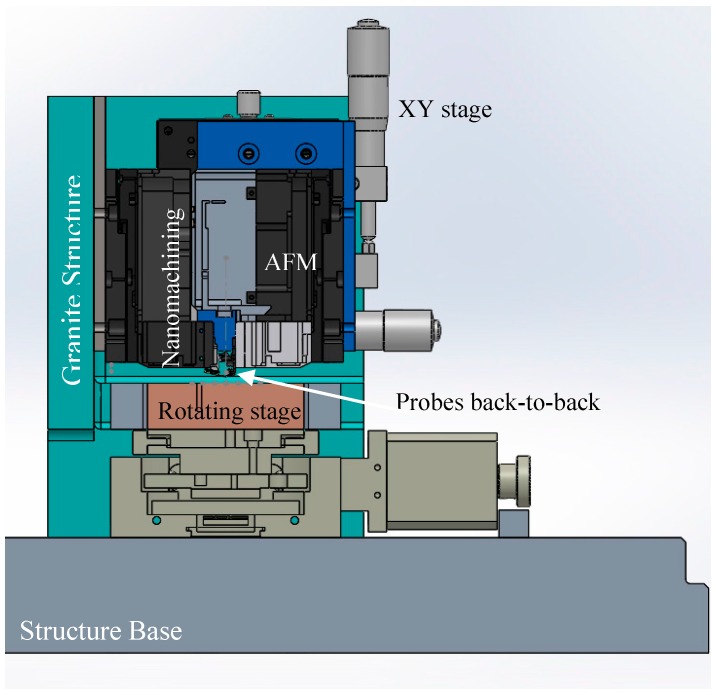
Cross section of the nanomanipulator with in-process AFM inspection.

All components are controlled based on the control architecture described in Figure 3. The highlighted colorful controllers are mainly for in-process inspection. Some vibrations can be noticed of the AFM and cutting probes due to an environment minimized by isolating the nanomanipulator from the ground vibration through a controlled damping table.

The focus of this paper is on the in-process inspection zone. An AFM is mounted on XY precision stage (Figure 3) with locks for each axis, so it is possible to adjust the probe height by a few millimeters to accommodate various part sizes. The piezoelectric scanner for nanomachining is mounted close to the AFM on a separate platform that is firmly mounted of the supporting platform. A space under the probes is allowed to receive the rotating table and to place samples under the two probes when needed.

**Figure 4:**
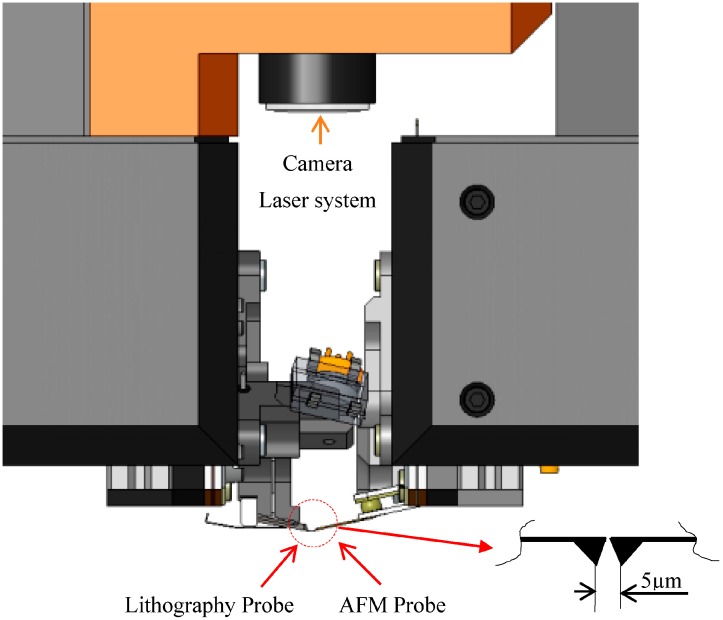
Position of AFM and lithography probes on the actual configuration.

**Figure 5:**
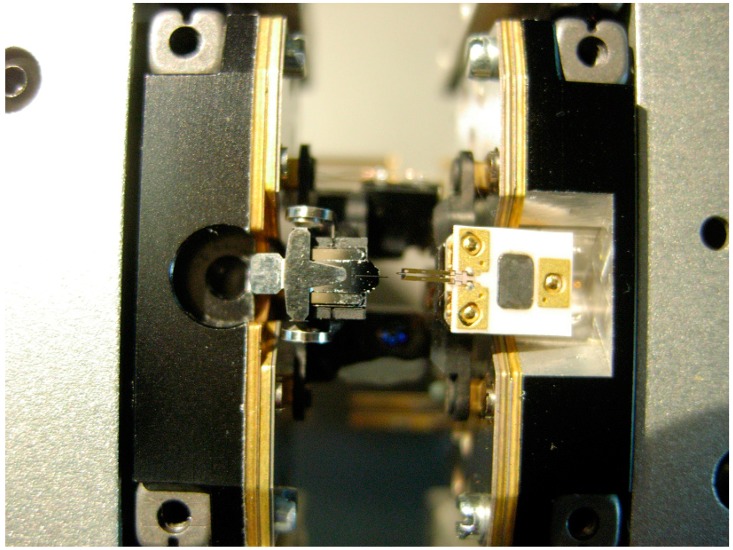
Bottom view of back-to-back probes (left: AFM probe NCLR-10 cantilever, right: Lithography: A-probe (Akiyama probe) cantilever).

The two AFM and lithography probes are mounted back-to-back with physical distance adjustment set to 5 µm as a security distance to avoid any crash between the probes. The two heads were little modified with covers removed and with little adjustment of the piezoscanner. Only one top view camera will be available for both systems as shown in Figure 4 for positioning. The detailed positions are shown in Figure 4 and Figure 5 where the distance between the probes is 5 µm. The probes can safely move horizontally without crashing against each other. The sample to be tested can be adjusted underneath the probes using one axis of the table.

**Figure 6:**
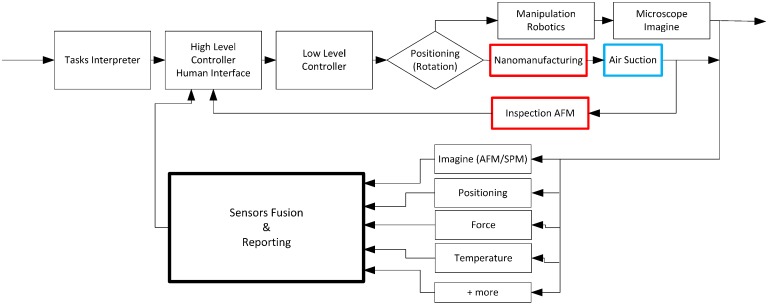
Overall control architecture for the manipulator.

The AFM inspection is carried out by a Nanite AFM scan head with a maximum scan range of XY 110 µm × 22 µm needing standard AFM cantilevers, while for the lithography, a scan head Nanite SH A110 having similar range of measurement as the AFM head is used in front. The AFM is controlled by SPM200 controller while the lithography head is controlled by SPM100 with speeds of up to 60 ms/line. Hence, both heads are independently operated but working under one script when both operations of lithography and in-process are in progress (Figure 6 and Figure 11). The overall control architecture is shown in Figure 6 where the both operation of lithography and inspection are operating in a loop, taking commands from high level control planned by human interface. The sample is controlled in position under the lithography and AFM probes.

**Figure 7:**
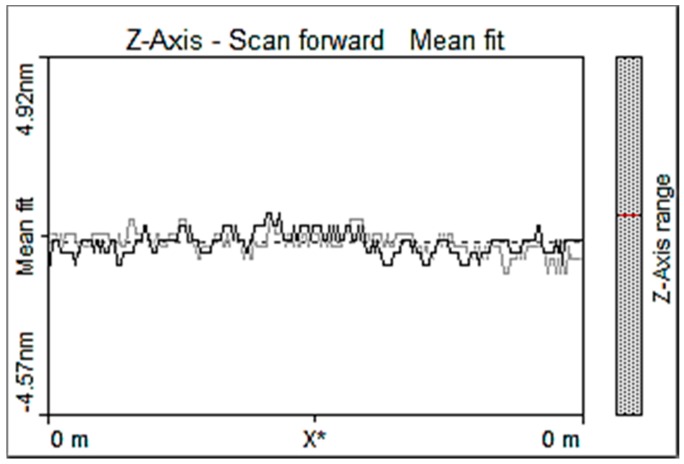
AFM tip vibration measurement.

A probe stability test on the AFM probe has been carried out to measure vibrations of the probe tip as shown in Figure 7. The average amplitude of vibration is less than 1 nm depending on the probe bending stiffness.

### 2.2. Nanomachining Modes and Inspecting Configurations

**Figure 8:**
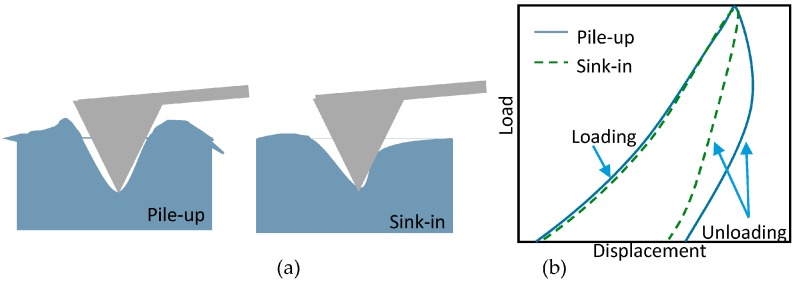
(**a**) Pile-up and sink-in; (**b**) load-displacement curves for elastoplastic material and viscoelastic and plastic response on soft material.

**Figure 9:**
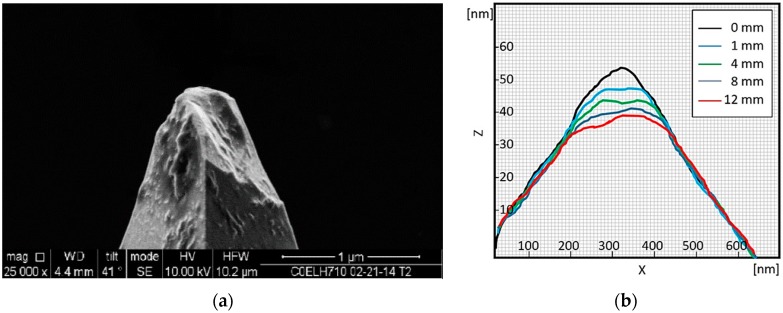
(**a**) Probe tip wear; (**b**) Example of wear rate at the tip.

Nanolithography using an AFM-like probe can be carried out by mechanical and electrical means. The first method needs a high flexural force, or a lateral force from the probe tip on the surface, the second method needs a high biased voltage is used to oxidize local surface. The scratching on the polyimide film is processed by an AFM (Figure 9) probe composed of a microscopic cantilever with a specific flexural stiffness and resonant frequency, and a tip attached to the cantilever known by its material with associated properties and dimensions e.g., apex radius, aspect ratio, hardness, and stiffness. Two types of scratching results can be obtained on the material. They can appear as either pile-up, or sink-in as shown in Figure 8a. The latter process results in neat grooves. To distinguish between the two methods, two different loading behaviors are governing as it can be seen from Figure 8b. For each type, the load applied on the probe has its own profile where the loading is similar for both but the unloading is different.

Irrespective of the desired mode described previously, the AFM probe tip will be worn progressively as long as it is in sliding contact with the opposite surface. Wear has been discussed in [12] where the contact radius of the tip can degrade by increasing its radius in two phases; the first one will be very fast with immediate increase of the radius when the tip breaks, the second one takes a longer time depending on the scanned distance. Figure 9a shows an example to probe tip wear with wear rates examples. It is required, depending on the mode of nanomanufacturing, to compensate for this wear and for other misadjustments. Figure 9b shows tip shape degradation after several passes from 1 mm to 12 mm. the initial shape is shown at 0 mm. The wear range can go up to 20 nm for continuous scan over 12 mm pass. The trajectory length used in this paper was less than 1 mm, with very good probability to keep the tips in their initial shapes.

### 2.3. In-Process Inspection

The inspection and characterization at nanoscale is carried out using AFM/STM head from Nanosurf with a probe. Nanomachining is also facilitated by a piezoscanner with independent controlled probe assembled back-to-back to the inspection AFM probe. One is an inspection probe, the second one is a processing, i.e., scratching probe. The two probes are located back-to-back within a safe constant distance of 5 µm and having a scanning range of a 100 µm.

**Figure 10:**
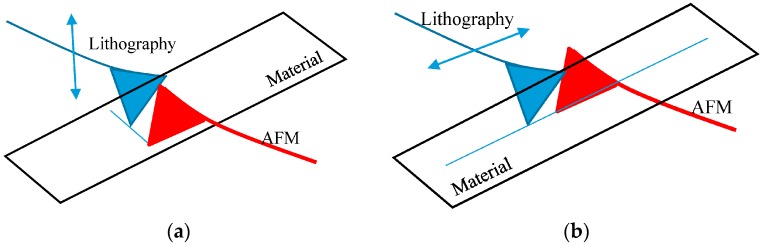
Two configurations of the in-process inspection. (**a**) Flexural mode (back-to-back); (**b**) Lateral mode (side-to-side).

The inspection probe configuration can follow the lithography probe depending on the mode selected for the lithography by being back-to-back, or, close lateral (side) if the lithography probe uses its lateral deflection that is higher than the bending. Figure 10 shows the two possible configurations. Only the back-to-back configuration is discussed in this paper.

## 3. Nanomachining and In-Process Inspection Experiments

Several tests have been carried out to trigger proper nanomachining and to observe the benefits of having back-to-back probes. The lithography tests included three probes with different tips—i.e., triangular, conical, and isosceles—that were running at various speeds and applied voltage on the same material sample that is available commercially.

**Figure 11:**
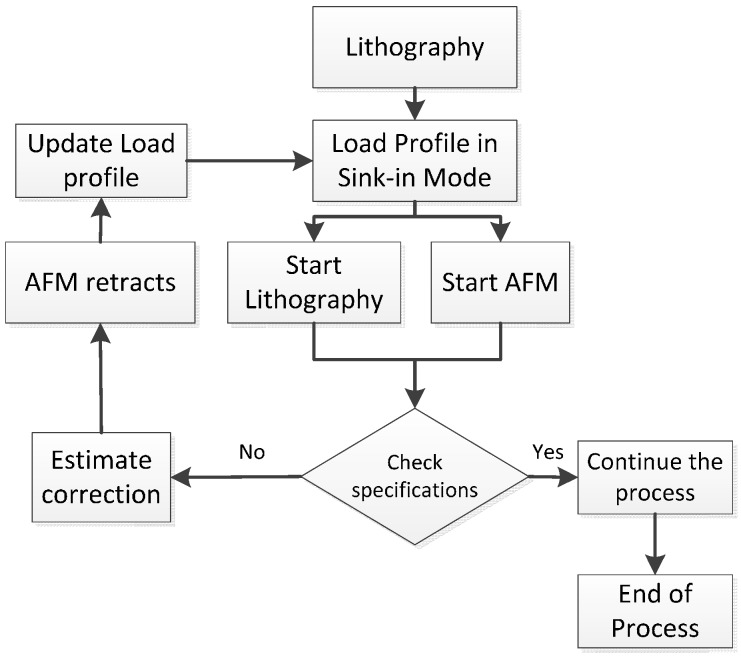
In-process inspection script.

Since the results were not too different, two tests have been selected for this current work after extensive trials; controlled polyimide depth scratch and series of manufactured grooves. The purpose here is to show in-process inspection. The following script shown in an algorithm was used in Figure 11.

**Figure 12:**
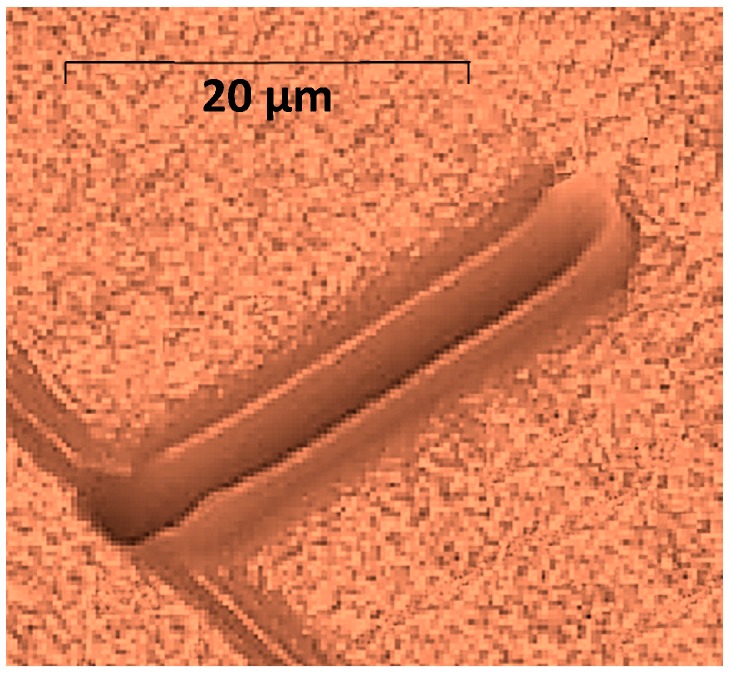
Scratch on polyimide film using 10 µN force (left to right).

An operated linear scratch was carried out on polyimide film with a normal force of 10 µN (Figure 12). The probe had a stiffness of 18.5 N/m with resonant frequency of 220 kHz. The probe length was 110 µm equipped with a Si tip having apex radius better than 10 nm. The specified depth of scratch was 150 nm but soon it was realized that the depth was at only 135 nm average. This has been corrected automatically towards the remaining 10 µm to 150 nm as it can be shown in Figure 12.

**Figure 13:**
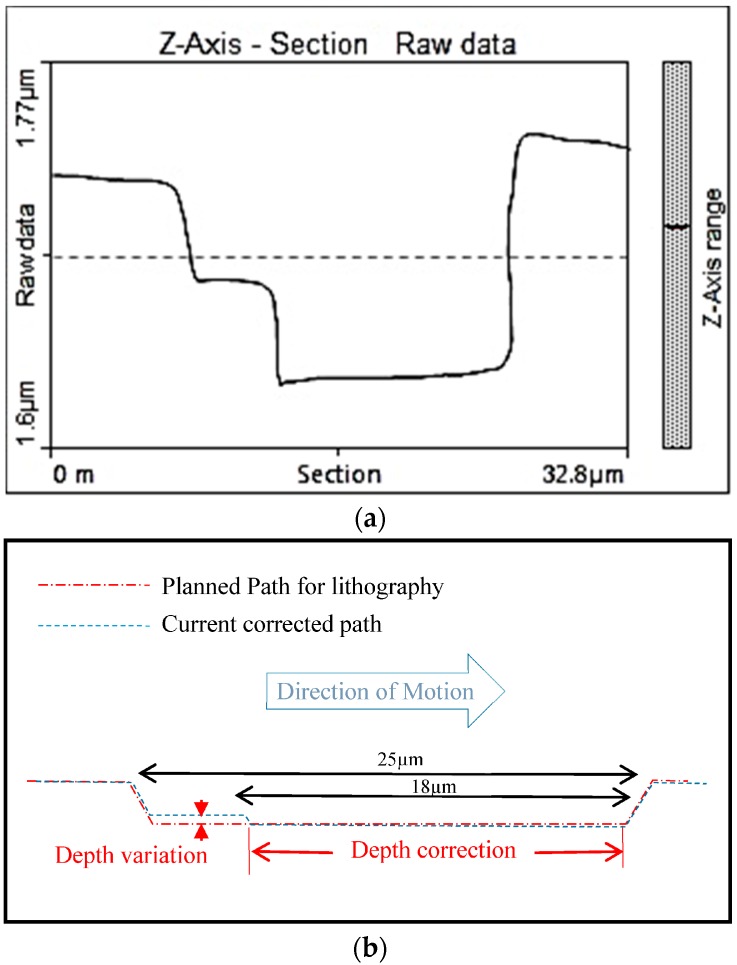
(**a**) Controlled lithography process on polyimide film (not to scale); (**b**) detailed process.

The inspection of the scratch shown in Figure 11 was progressing as the lithography probe was moving by a gap distance of 5 µm as this was the gap when positioning the probes back-to-back. The correction introduced here was triggered when the AFM probe reports the incorrect depth (Figure 13), hence parameters on the lithography can be updated to cut more in-depth by the spotted difference. If it is too late (always by 5 µm at least) because of this gap, then the lithography probe is requested to return to where the defect has been detected and a correction will be then implemented as it is seen in Figure 13a.

**Figure 14:**
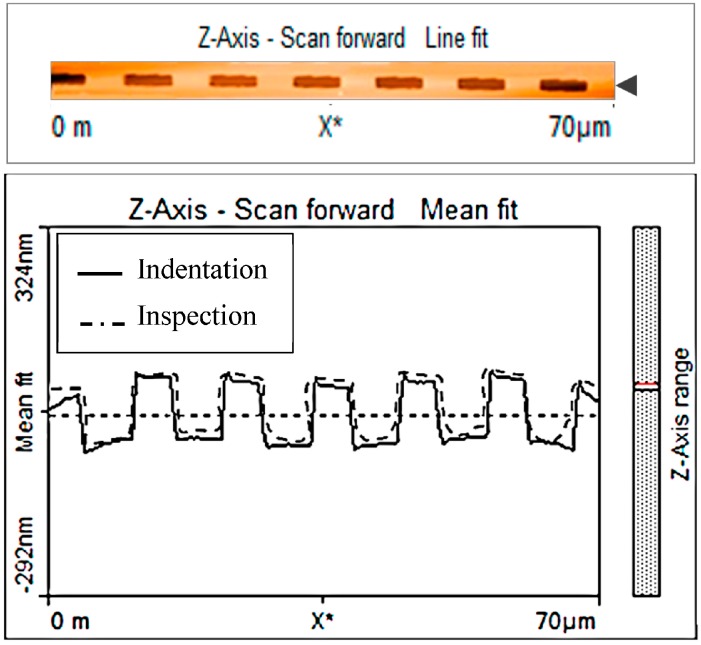
(**Top**) Indented material; (**Bottom**) measurement in-process using back-to-back probes.

In the next test, a nanolithography process was executed on a series of PMMA material samples in sink-in mode as introduced previously. A series of grooves were indented over 70 µm length with an average depth of 10 nm (Figure 14 top). In-process measurement by AFM probe in tapping mode compared to the probe executing lithography has shown little expected discrepancy (Figure 14 bottom) but is still in good agreement with the initial request.

## 4. Conclusions

An in-process AFM based inspection system using back-to-back probes has been developed, tested and presented in this paper. This proposed sensing configuration conserves the AFM measurement quality of the manufactured features; compensates for errors generated by various sources, e.g., continuous degradation of the probe tip; and obtains features that are nanomachined to specifications.

The nanomachining with in-process inspection has been proven to be very smooth and to have a unique advantage of error compensation that can be either manual or automatic while monitoring the process. The distance between the two tips, although constant, will always delay the AFM tip to trigger any correction. Reducing this gap by selecting rectangular tips, for example, will improve the quality of lithography to specifications. The in-process inspection will help also to better understand nano-manufacturing processes.

The next focus of study would be to consider side-to-side probes position and to implement an automated correction within the manufacturing process considering the initial lithography specifications as a reference.
